# “Natural Laboratory Complex” for novel primate neuroscience

**DOI:** 10.3389/fnint.2022.927605

**Published:** 2022-10-05

**Authors:** Atsushi Iriki, Antonella Tramacere

**Affiliations:** ^1^Laboratory for Symbolic Cognitive Development, RIKEN Center for Biosystems Dynamics Research, Kobe, Japan; ^2^Department of Philosophy and Communication Studies, University of Bologna, Bologna, Italy; ^3^Department of Cultural and Linguistic Evolution, Max Planck Institute for the Science of Human History, Jena, Germany

**Keywords:** longitudinal studies, socio-ecological studies, natural(istic) environment, advanced technologies, remote infrastructure, primate welfare, dilemma and trade-offs

## Abstract

We propose novel strategies for primate experimentation that are ethically valuable and pragmatically useful for cognitive neuroscience and neuropsychiatric research. Specifically, we propose *Natural Laboratory Complex* or *Natural Labs*, which are a combination of indoor-outdoor structures for studying free moving and socially housed primates in natural or naturalistic environment. We contend that *Natural Labs* are pivotal to improve primate welfare, and at the same time to implement longitudinal and socio-ecological studies of primate brain and behavior. Currently emerging advanced technologies and social systems (*including* recent COVID-19 induced “remote” infrastructures) can speed-up cognitive neuroscience approaches in freely behaving animals. Experimental approaches in natural(istic) settings are not in competition with conventional approaches of laboratory investigations, and could establish several benefits at the ethical, experimental, and economic levels.

## Introduction

Neuroscience has made tremendous advancements in understanding the biological mechanisms and processes of the human mind. A considerable part of these advancements has been achieved through research in animal models, including non-human primates (NHPs). Notwithstanding, in the last decades, in most Western countries, NHP experimentation have faced practical challenges and ethical restrictions, largely due to difficulties of keeping pace with economic costs of NHPs facilities, and ethical concerns that primates experience suffering similarly to humans (EU Commission, 2013; Lankau et al., 2014).

Meanwhile, rodents have become (and very likely will continue to be) the commonest animal model in neuroscience (Homberg et al., 2017), often replacing NHPs in experiments about mechanisms of cognitive functions. One of the reasons for the widespread use of the rodent model in neuroscience comes from rodent cognitive complexity. Astonishingly complex cognitive capacities have been recognized in various species of rodents. Rodents are very skilled problem-solver, have highly developed motor capacities and show a variety of social and non-social cognitive mechanisms (Davis, 1996; Schweinfurth, 2020).

Additionally, pragmatic reasons make rodents very much investigated in neuroscience labs. Mice and rats are easy to breed and to test in laboratory settings and have very flexible behavioral habits, which raise relatively few concerns from an ethical and practical standpoint (Hickman et al., 2017).

Many have pointed out however that the rodent model is limited when explanatory targets of the human mind do not find adequate counterparts in the rodent anatomy and physiology (Roelfsema and Treue, 2014; Camus et al., 2015). Differences between rodents and humans become even more problematic if one considers the recent pragmatic turn in cognitive science (Engel et al., 2016), for which mental functions and dysfunctions are not exclusively the result of internally regulated brain mechanisms, but also of brain–body and brain–environmental interactions. As the mind is progressively understood in terms of bodily rules and situated aspects of environmental interactions, approaches that abstract from the specie-specificity of these aspects in the target animal models become outdated.

Every animal species possesses characteristics and idiosyncrasies that are difficult to generalize to other animal species. Therefore, experimentations with animal models will always show some range of limitations when the goal is generalizations of mechanisms to humans. No animal model could ever exactly recapitulate human cognitive capacities. However, the evolutionary relatedness, as well as the similar rules of interactions between biological and social factors in complex environmental niches (Camus et al., 2015), often make NHPs outstripping candidates for inquiring about processes and mechanisms of the human mind.

If we agree with this assumption, then we face a *dilemma* regarding the role of primates in neuroscience; that is, while phylogenetic proximity with humans dictates primary relevance of NHPs, the same proximity produces ethical concerns and pave the way to difficulties in regulating research practices and regulations. Taking primate research dilemma seriously implies that neuroscience needs to balance the trade-offs between ethical considerations and practical benefits for advancing the understanding of the evolutionary precursors and basic mechanisms of the human mind. The dilemma also implies that, because of the standard of welfare that we want to grant to NHPs in neuroscience research, any novel proposal for NHP experimentation shall take into consideration cost-benefits trade-off for all partners, including the primates (Carvalho et al., 2019).

In this article, we intend to lay the ground for such a project, by advancing the conceptual basis and technical requirements for novel strategies in primate experimentation. We start by briefly overviewing pros and cons of neuroscience research with rodent and primate models to show that both models can be instrumental to address different, but complementary research questions. Then, we propose a novel endurable setup of NHP experimentation, as an ethically valuable and pragmatically useful strategy for cognitive neuroscience and neuropsychiatric research. Specifically, we propose *Natural Laboratory Complex* or *Natural Labs*, as a combination of indoor-outdoor structures for studying free moving and socially housed NHPs in natural(istic) environment.

We argue that cognitive neuroscience approaches in freely behaving animals could increment productive exchanges between existing conventional approaches of laboratory investigations. We further make a series of considerations regarding how *Natural Labs* could establish beneficial ethical, financial, and legal trade-offs for leading NHP neuroscience to the next stage.

## Animal models in neuroscience

### The rodent model

Mice and rats belong to the order Rodentia, which is the closest to order Primates in the class Mammalia. Rodents possess homologous features with human and NHPs, and numerous similarities at the anatomical, physiological, and organizational level (Ellenbroek and Youn, 2016).

Many neurocognitive mechanisms which are common to both primates and rodents have been discovered or delved into through mice and rats research in the last decades, including fine-grained mechanisms of manual control, vocal plasticity, and navigation capacities (Davis, 1996; Arriaga and Jarvis, 2013). One obvious example of the latter is the discovery of place cells in the hippocampus of rodent which has opened new lines of research on memory and spatial cognition (O’Keefe and Dostrovsky, 1971; Thompson and Best, 1989).

The diffusion of the rodent models is not exclusively due to neuroanatomical and behavioral rationalizations, but also to pragmatic reasons. Mice and rats have characteristics which make their colonies cost efficient to maintain. In fact, most rodents are small, are easily housed in laboratory settings, have short gestation time and large numbers of offspring, rapid development to adulthood and short life spans (Bryda, 2013; Hickman et al., 2017).

Further, many novel techniques of intervention, such as tools for genetic modified engineering, have been developed in rodents, because the rodent model is considered a good compromise among animal species which approximate human brain complexity and where these experimental procedures are more ethically acceptable (Baker et al., 2020). Through novel techniques, several molecular factors associated to specific normal or abnormal cognitive states in human beings have guided mechanist studies in rodents (Kaiser and Feng, 2015). As a consequence, pioneering advanced technologies in genome engineering have facilitated the creation of genetically manipulated rodent models of various psychiatric disorders, enabling investigations of underlying biological mechanisms (de Hamilton, 2013; Homberg et al., 2016).

Despite the advantages of rodent experimentation in pre-clinical and clinical research, findings from rodent models turned out not to be necessarily generalizable into humans and have resulted in failure to develop treatments of targeted mental and psychological disorders (Jennings et al., 2016). This has discouraged some pharmaceutical companies to invest in neuropsychiatric research which was largely based on earlier experimentation with rodents, and to downplaying associated drug trials (Ayesha, 2021). The causes for these results are likely various and heterogenous, see here for some recent analyses of the question (Fernández, 2019).

**FIGURE 1:**
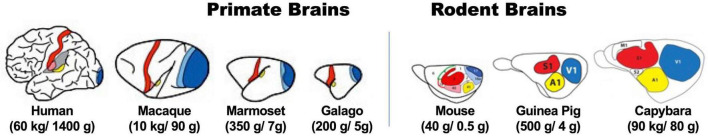
Diagrams illustrating different organization principle between primates (left) and rodents (right) by comparing brain structures of species with different body weight and brain sizes within respective mammalian order. Colored areas in brain illustrations indicate primary sensory (red for somatosensory, blue for visual, and yellow for auditory) areas in representative extant primate (left) and rodent (right) species of body (first numbers in brackets) and brain (last numbers in brackets) sizes (adopted and modified from Krubitzer, 2009; Krubitzer and Dooley, 2013). Note the difference in proportion of these primary areas and association areas (in white) in different sized-brain between primates and rodents. (*Ref.* color symbols not directly related to the scope of this article, but illustrated in the original figures: green, motor area; pink, secondary somatosensory area; light blue, secondary visual area.)

It is difficult to exclude, however, that differences in brain structure and functions between human and mice could be partially responsible for the hurdles of generalizing neurocognitive mechanisms from rodents to humans (Stephan et al., 2019). Potential differences regard the number of neurons in the brain, organization of the cerebral cortex (Figure 1), resulting patterns of intracortical connections, and the actions of neurotransmitters and neuro-modulatory pathways (Herculano-Houzel, 2012). These differences might well hinder straightforward generalization of rodent neurobiological mechanisms to humans, despite cognitive and behavioral phenotypes look similar.

Another factor to consider is that in humans the emergence of psychological and cognitive traits, and vulnerability to disorders, is conditioned upon interactions of bodily and developmental factors across individuals, which include the specificity of the bodily plan and organization, and the complexity and richness of the socio-ecological niche. These aspects make human mental functions substantially different from caged laboratory animals. These differences are particularly exacerbated for biologically controlled pure strain rodent models which are bred in caged-based facilities.

In sum, significant differences in the anatomical, brain and affective systems in small experimental animals and humans provide strong limitations for modeling and studying psychological and behavioral dysfunctions in mice and rats. Therefore, basic and applied investigations of psychological and cognitive functions which are mostly exclusively conducted in rodents can be problematic.

Many previous contributions have analyzed pros and cons of rodent research, and we will not repeat it here. We are aware that none of the evidence listed above can constitute a conclusive argument of what rodent research in neuroscience can or cannot offer for generalization of results to humans. Consider however that our aim here is not to demonstrate that the rodent models is *per se* limited in cognitive neuroscience research. With this admittedly brief analysis of potentialities and limitations of rodent research, our scope is only to point out that, despite critical advancements, cognitive neuroscience research in rodents is not alone sufficient for understanding the biological mechanisms and processes of the human mind. We thus think it is important to develop strategies to overcome experimentation restrictions in NHP and benefit of the NHP model in cognitive and behavioral research.

### The non-human primate model

Many neuroscientists have highlighted that the primate models are unparallel animal models in cognitive neuroscience and neuropsychiatry (Roelfsema and Treue, 2014; Zhou, 2014), which offer valuable mechanistic information for how the human brain works, and for potential psychiatric vulnerabilities.

One obvious aspect regards similarity of brain organization between most primate species and humans. Whereas rodent species with significant difference in brain size have the same relative size ratio of cortical areas, primate species with larger brain have higher numbers of associative cortical areas, with consequent more varieties of intracortical connections leading to drastic expansions of higher-order functional properties (Herculano-Houzel, 2009, 2012; Figure 1). This makes at least some NHP species optimal models for investigating neural mechanisms of the human mind.

Further, various neurocognitive mechanisms are common to humans and monkeys, while different in rodents. These include object recognition capacities, working memory, fine-grained processes of decision making, the frontal-parietal processing of visuomotor behavior, the neural basis of quantification skills, the complexity of social learning, and the interplay between conscious and unconscious processes, just to nominate a few (Capitanio and Emborg, 2008).

Primate experimentation facilitates studies of the functional properties of single or populations of neurons involved in behavior grounded on human-specific bodily plans, and which contribute to the development of new treatments. Consider for example, the discovery that electrical stimulation of subcortical structures alleviates many of the symptoms of Parkinson’s disease (Collier et al., 2005). These symptoms are linked to the primate-specific motor organization and are difficult to observe in species with different sensorimotor characteristics.

Another case regards the discovery of mirror neurons in the premotor cortex of the macaque monkey (Ferrari and Rizzolatti, 2015), which inspired new experimental hypotheses in Autism and Schizophrenia, and new therapeutic intervention in patients e.g., suffering of facial paralysis and Mirror-Touch synesthesia (Banissy et al., 2009; de Hamilton, 2013). In all these cases, the specific relationship between primate brain structure to bodily and socially situated functions have been crucial to uncover mechanisms generalizable to the human mind, paving the way to critically therapeutic perspectives.

That embodied aspects of human mental functions and mechanisms can be efficiently investigated in NHPs does not imply that investigating these aspects in other animal species is not instructive. For example, mirror neurons have also been inquired about in rodents (Viaro et al., 2021) and birds (Mooney, 2014; Tramacere et al., 2019) and linked to respectively manual and vocal behavior. While the comparative approach in neuroscience is fruitful to inquire into the biological bases of cognitive variations, it does not dispense of the lack from primate experimentation. Rather, cross-species comparison between non-primate species and humans requires also analyzing the NHP brain connected to primate-specific evolutionary variations in embodied and socio-ecologically situated cognition and behavior.

From an anatomical point of view, primates possess homologous bodily structures with humans making easier to analyze certain functions related to posture, facial expression, gaze, and manual gestures, which result from the use of the body in interaction with the physical and social environment. Consequently, the morphological commonalities also allow to inquire the role of environmental and social interactions in the emergence of cognitive functions and dysfunctions (Chang et al., 2013).

Behaviors such as manual dexterity and tool-use (Iriki, 2006), communication based on the mobility of facial expression (Tramacere and Ferrari, 2016), important aspects of social and individual cognition (O’Connell and Dunbar, 2005; Dunbar, 2009), the mechanisms of attention and visuomotor behavior (Rizzolatti et al., 2001; Peeters et al., 2009; Yang et al., 2016) are different among primate and non-primate animals.

Further, from a molecular genetic standpoint, the phylogenetic proximity of humans and NHPs give more guarantees that they will share more specific genetic mechanisms involved in neurophysiology, behavior, and susceptibility to disease. As NHPs and humans are part of the same phylogenetic lineage, NHPs have largely shared gene maps, over other mammalian orders, and therefore can contribute to analyze complex gene-gene or gene-environment interactions at the basis of human neurophysiological processes (Rogers and Gibbs, 2014).

To conclude this analysis with a further example, consider a recent study from Yoshida et al. (2016) with macaque monkeys. The authors serendipitously noticed that one individual of the primate group presented a set of behavioral manifestations that were consistent with autistic-like phenotype in human beings. These manifestations included primate specific affective behavior, such as lack of grooming and facial interactions, and presence of nail-biting stereotypes.

Although grooming in human beings vary across cultures (Jaeggi et al., 2017), reciprocal cleaning practices, together with facial exchanges and stress-related nail biting are primate (including human)-specific behaviors that cannot be observed in animal species with different bodily and socio-environmental components. We take this study as an example to suggest that the primate model is important to understand the emergence of cognitive mechanisms and functions that are common to human and NHPs and illustrate how they are involved in the emergence of psychiatric conditions.

Note however that NHP experimentations have disadvantages in cognitive neuroscience and neuropsychiatry. NHPs are larger than laboratory rodents, thus experiencing discomfort when living in cages, especially because social interactions and active exploration of the environment are essential to their development and psychological wellbeing (Conlee and Rowan, 2012). Further, as previously mentioned, NHP experimentation produce concerns from an ethical point of view, because of evidence that primate species experience suffering in a similar way to humans.

Among the disadvantages of primate experimentation, technical challenges must also be considered. Not all investigative tools are easily utilized in the NHP models. For example, optogenetics, a genetically coded channel technique that allows high temporal and spatial resolution of cortical and cerebellar brain circuits, have faced a slow progress in NHPs (Galvan et al., 2018). The same is true for other techniques, such as for example chemogenetics (Raper and Galvan, 2022), and transgenic tools (Coors et al., 2010), which are thought to produce unacceptable suffering and pragmatic difficulties in monkeys.

In the next sections, we will capitalize on the pros and cons of primate and rodent research to propose a novel strategy of primate experimentation that can increase productive exchanges between experimental sub-fields conducted with different animal models.

### Optimizing cognitive neuroscience research with animal models

Research on animal models depends on many factors, such as the nature of the questions and the characteristics of the species. However, based on the pros and cons of rodent and primate models briefly depicted thus far, some generalizations are possible. By considering both characteristics of the models and of the current practices of research, rodent and primate research can serve different (and perhaps complementary) aims in neuroscience and neuropsychiatry.

Many primate species share analogous principles of brain organization with humans, homologous body plan and similar rules of interactions between social and biological (i.e., genetic) factors. Further, human psychological and cognitive traits, and vulnerability to disorders, result from human evolutionary history, and are conditioned upon the complexity of the environmental and social world. The potential of primate research, and the limitation of rodent experimentation, thus critically depend on the importance of the ecological, embodied, and situated aspects in the development of the human mind.

When the overarching goal is to understand the basic mechanisms of the human mind and the target of research is primate-specific neurocognitive factors, NHPs are likely to be optimal candidates for cognitive neuroscience and neuropsychiatry. In the perspective of a basic, pre-clinical and clinical neuroscience which must take into consideration the social, bodily, and environmental context of brain development and function, research in NHP is going to be going to provide information that are not possible to achieve through rodents.

Research with rodents (and eventually with other non-mammals) can be used to inquire into conserved evolutionary mechanisms or convergent functions that are realized through instantiation of different brain mechanisms. In addition, based on the genetic, anatomical and socio-ecological similarity between human and certain NHP species, NHPs shall be investigated in a way that is deeply embedded in and integrated with NHPs’ naturalistic settings, to inquire into the primate brain and behavior in an ecologically, longitudinally, and socially valid approach.

**FIGURE 2:**
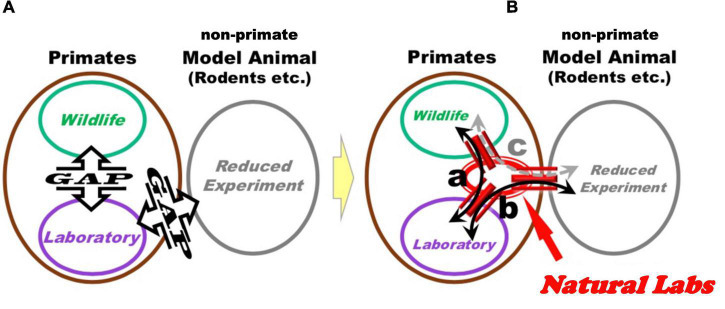
Through the implementation of the Natural Laboratory Complex by the present proposal, it may be possible to increment exchanges between different research fields. **(A)** Schematic representation of current research situation, depicting three sub-fields of primate research: wildlife studies, laboratory experimentations (on the left), and non-primate animal model reduced experimentations, rodents or otherwise (on the right). Communication and exchange between these three components are not systematic and left to personal exchanges among individual researchers. **(B)** “*Natural Laboratories Complexes*” can provide bridges between existing experimental sub-fields. Specifically, investigation of behavior, biological samples and brain activity in *Natural Labs* can be instrumental to (a) design classical laboratory experiments under controlled conditions, which can inform additional experimental designs in *Natural Labs*; (b,c) investigate analogous mechanisms in primate and non-primate animal models to identify potential conserved functions, and use results of these experiments to inform analysis and models related to findings obtained in *Natural Labs*.

Experimentation of NHPs in naturalistic settings is currently an underdeveloped field of research, which may fill existing *gaps* between classical lab research in neuroscience and wildlife investigations, where these gaps can be defined as a lack of harmonization and systematic exchanges between respective research sub-fields (Figure 2A). Laboratory neuroscience, as a branch of the biomedical field, normally tries to identify reproducible and generalizable causal brain mechanisms, by decomposing structurally or functionally the target mental phenomena through controlled conditions. In contrast, wildlife studies rely on inquiring into the diversity and individual uniqueness of animal lives in natural environments, through *descriptive* observations of their behavior without intervention.

**FIGURE 3:**
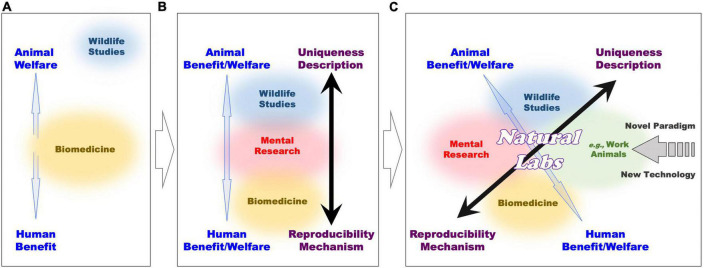
Comparative representation of ethical and experimental frameworks in various domains of animal research, along progressive development of interactions among multiple sectors (see Box 1 text for further explanations).

The laboratory and wildlife approaches retain important differences also at the ethical level (see Figure 3A and Box 1). Whereas laboratory statistically controlled conditions are instrumental to the quality of research practices, they also automatically imply that animals are subjected to them. These conditions include isolation from peers and deprivation of social interactions, living in captivity, research training with repetitive behaviors which are often not intrinsically rewarding, and so forth. In contrast, naturalistic and often descriptive observations of animal behavior normally require no intervention and operate easily by respecting animal welfare.

**BOX 1 Harmonization of ethical and research gaps.**

Animal welfare and human benefit are in opposition depending on the field of research. Different fields often require different approaches to animal rights and welfare.

Figure 3A illustrates separation of methodologies between the existing biomedical (orange circle; middle) and the wildlife studies (blue circle; top). Gaps exist between these studies (blue arrow), because biomedicine maximizes human benefits, while wildlife studies are more respectful of primate welfare. Therefore, large efforts are put to balance and compromise between animal welfare and human benefit with ethic regulations and guidelines, which consider harm-benefit trade off and 3Rs (replacement, reduction, and refinement) to minimize suffering of laboratory animals. This situation changes once cognitive neuroscience and neuropsychiatric research (pink circle; middle) comes to interplay (Figure 3B), because it can lead to bridging wildlife (blue circle; top) and biomedical (orange circle; bottom) studies.

By implementing primate research in naturalistic settings (green circle; with similarity with domesticated and working animals, i.e., nursing, companion, assistance, guide-animals, etc.; and conditions), the open field of primate cognitive neuroscience and neuropsychiatric research (*Natural Labs*) emerges to be carried out at the intersection of the existing research sub-fields (Figure 3C). Here, the use of a variety of novel (minimally invasive) techniques can grant the mechanistic approach of biomedical research, while primates living in natural settings can benefit of conditions that are normally proper to descriptive wildlife studies. In this way, neuroscience research in *Natural Labs* overlaps with both formerly separated wildlife and biomedical research, and reduces the “gap” between animal welfare and human benefit. Through *Natural Labs*, former polarizations between primate and human welfare, and mechanist and descriptive approaches become continuous. The application of new technology, subserving this new field can affect also the other field of research to trigger a novel (methodological and ethical) paradigm.

We think that in between the gaps at the ethical and research level resides a strategic potential to integrate relevant research fields through primate experimentation. Specifically, we propose Natural primate labs as a combination of open and enclosed spaces, where NHP populations can live in group and move freely in a setting of (or resembling) their natural habitat. These spaces would be open to the use of non-invasive or minimally invasive methods of brain, behavioral and bodily markers investigation.

The aim of these *Natural Labs* is to capitalize on the descriptive approach of wildlife studies, by focused observations of NHP behavior in naturalistic conditions (Figure 3B). At the same time, the mechanistic approach to specific NHP cognitive functions through the use of brain techniques can allow holding some of the benefits of the laboratory approach. These two elements (descriptive and mechanistic) may put primate *Natural Labs* in a fruitful communication with the traditionally opposite frameworks of wildlife and laboratory research (Figure 3C).

Primate *Natural Labs* can allow to fruitfully connect different research approaches through the use of a range of techniques to investigate primate brain, behavioral and bodily manifestations (which we will describe in the next section). The combination and flexible integration of novel strategies of primate experimentation in *Natural Labs* becomes even more critical if we consider that, when released into naturalistic environment, animal models start exhibiting much richer and more complex behaviors than in cages [see for example, the documentary of Berdoy (2017)] (Bottom et al., 2020). Therefore, quantitative comparisons between free-moving and captive primate and non-primate animals, could inform focus on appropriate target mechanisms of behavior.

Finally, NHP experimentation needs to be adjusted for a broad range of neuroscience investigation, that could complement the research that is already done in laboratories with human, primate, and rodents, and in the wild with primates. This is where the integration with classical laboratory research, in both primate and non-primate models, become important and can pave the way to the investigation of phenomena that could not be displaced in classical research settings.

## Novel strategies for primate experimentation

Because in *Natural Labs* primates live freely in naturalistic spaces, the realization of these structures could allow for the collection of data in a multimodal, longitudinal, and ecological perspective, leading to analysis of various types of behaviors and their underlying neurobiological mechanisms.

In this settings, close collaboration with husbandry and research personnel as equal partners is important to conduct breadth of studies from observational ones (to observe and record animals’ behavioral manifestations) to neurocognitive investigations (to approach animals and perform cognitive, neural and biological examinations), because animal hosted in these structures need appropriate cleaning, space and nutrition measures. Therefore, husbandry efforts to secure behavioral manifestations will necessarily become continuous with research practices, and research endeavor will become a part of husbandry, thus equally amalgamating both sectors.

The idea of making husbandry continuous with research efforts is partly similar to what William Conway called “interactive management” of zoos and natural habitats (Conway, 1995). Conway defined interactive management as a strategy for both species and habitat preservation that relies upon the coordination of species living both in a combination of natural and artificial settings. Interactive managements usually combine the resources of wildlife managers with those of biologists working in zoos to ensure that animal individuals are provided with opportune conditions, and do not suffer for abrupt environmental or habitat changes.

Our proposal of a novel strategy for primate research in the wild or in natural-like settings has a different aim but rely on a similar rationale, because primate species in *Natural Labs* shall live in mixed natural-artificial conditions for research purposes, and not for conservation efforts *per se*. Although the type of primate research that we are proposing can lead to conservation advantages (which we will touch upon later), it primarily concerns an ethically and pragmatically more sustainable way to conduct neurobiological and ethological research on our closest relatives (also see Box 2).

**BOX 2 Rationale and examples of *Natural Labs*.**

The concept of *Natural Labs* relies on the awareness that the traditional view of protecting animals by letting them live in a vast self-sustaining “wild” need to be rethought. We must begin to face the reality of a world that is and will remain vastly altered by human activities. Consequently, taking actions for ensuring that many more wildlife communities can survive if they are intensively cared as a form of “megazoos” (Conway, 1995) could be more efficient than being stacked in searching a wildlife for animals untouched from human intervention. The interactive management proposal is thus a wide way for zoos, working with local communities to support the survival of species under risk of extinction.

The idea of *Natural Labs* for primate experimentation can be framed in this perspective. One obvious example of *Natural Labs* is the *primate bio-park*. They are zoo-like spaces for hosting various species of NHPs, and where it is possible to collect multiple socio-behavioral, neural, and molecular information associated to cognitive phenomena in a longitudinal perspective, and in accordance with the application of novel technologies. NHP populations hosted in these bio-parks mimic natural population living in the wild and constitute a natural source of variability that may be critical for achieving mechanist models of cognitive and behavioral development.

The most similar examples of bio-parks we are here illustrating can be found in the Pongoland Wolfgang Köhler Primate Research Center, which operates in collaboration with the Leipzig zoo, or in The Apenheul Primate Park in the Netherlands. Both of them are zoos where visitors can see and interact with different species of primates, but they also house a research center, where different types of investigations are conducted. To make these bio-parks more similar to our proposal, they should be equipped with technologies for experimenters to inquire the primate neurobiology beyond the observation of behavioral aspects. Incidentally, genetic biodiversity in these parks may be guaranteed through periodic assisted reproduction in females, or through migration of male or female specimens, in accordance with the sexual behavior of the considered species.

Another form of *Natural Labs* would require that scientists conduct research in the primate natural environment. This would require the establishment of what has been called *hotel space* next to primate natural environment, where visiting researchers can carry out various types of experiments. Telecommuting approaches may be utilized to share information with scientists on site (Jennings et al., 2016).

We are referring to the types of novel technologies that allow the storage and transfer of information in real-time, such as Internet of things (i.e., a new paradigm in modern wireless telecommunication) (Atzori et al., 2010), IT based knowledge management (i.e., information technology system to enhance and organize knowledge) (Alavi and Leidner, 1999), and cloud-based Big Data processing for allowing cost-efficient exploration for voluminous data set (Fang et al., 2015). Alternatively, scientists could utilize telecommunication technologies (i.e., remote labs) from a separate geographical location, to remotely conduct real experiments at the physical location of the operating technology (Quesada-González and Merkoçi, 2017).

The rationale of the hotel space with remote technologies can be appealing also to investigate the number of NHP individuals living wildly or semi-wildly in countries, such as Japan, Singapore or anywhere close to primates’ natural habitat. In Japan, for example, several monkey parks are spread throughout the territory and are open to visitors. In Singapore, although not specifically for primates, there are many Safaris where animals live in wild-like environment and are managed by caretakers under international standards of animal welfare. These animals can constitute a further source for studying inter-individual variability at the behavioral level, and analyzing various biological samples (e.g., from the feces to the blood or the buccal mucosa).

Someone could object that close collaboration between husbandry and research personnel as equal partners might be difficult. This is because, in the existing animal experimentation contexts, conventionally trained researchers do not directly take care of the practical needs of their animal models, and animal care taking staffs are normally neutral about the scopes of the experiments, perhaps in order to avoid any implicit bias to affect on the scientific research outcomes using laboratory animals.

Coordinating the collaboration between husbandry and research sectors requires focused reflection and dedicated strategies, that go beyond the scope of this article. We would like to provide however one potential clue. In order to secure husbandry efforts for behavioral manifestations as a portion of research, and of research activities comprising a part of husbandry, strategies could be extrapolated from the mode of human commitments in collaborations with working animals (i.e., nursing, companion, assistance, guide-animals, etc.) in which caretaking and behavior analyses/control is undertaken as an integrated endeavor, in an environment somewhat resembles the laboratory conditions.

### Natural laboratory complex

In order to realize the proposed research strategies, studies should be designed to be conducted in structures comprising field sites in natural settings (***in situ* Lab-in-Nature**) and laboratories resembling primate natural habitats (***ex situ* Nature-in-Lab**), both of which are equipped with advanced technologies appropriate to observe and investigate NHPs’ brain, biology and behaviors. We describe the technologies that could be used in the Natural Laboratory Complex, together with how they could balance cost and benefits trade-offs at the ethical and economic level.

#### *In situ* Lab-in-Nature

Various types of technologies could be used in *Natural Labs* to investigate primate neurobiological and behavioral functions. Here, we elaborate on some examples to offer ideas of how these indoor-outdoor research space could be technically organized.

Firstly, a few deep learning algorithm techniques, each tailored to the research question, can be employed to estimate behavioral poses in monkeys interacting in the social group or during specific tasks (Nath et al., 2019; Lauer et al., 2022). These data could be integrated with fine-grained behavioral analysis in socio-environmental set-ups (Nishikawa and Kinjo, 2018), allowing investigation of previously unavailable ecological variations.

Fully non-invasive AI (artificial intelligence)-based motion capture of monkeys in the wild, deriving VR (virtual reality) and IT (internet technologies) for sharing and analyses of those motion data may be important technologies for integrative neuroscience investigations of *in situ* Lab-in-Nature. The social or individual behavior of primates could be recorded and analyzed by fixed-point live digital cameras installed at the field site and subjected to AI-based motion capture and 3D reconstruction using online deep transfer learning-based automated motion analysis technologies (Nath et al., 2019; Lauer et al., 2022).

Non-human primate behavioral datasets could be embedded (as avatars) in digitized environmental information (i.e., laser-scanned landscape, vegetation, meteorological conditions by remote-sensing devices, etc.) using VR technologies to comprise “*Real-time 3D Digital Zoo*” accessible to world-wide academic professionals for research purposes through IT-based cloud technologies, which have achieved prevalent development under current COVID-19 restrictions of inter-regional traffics. This set of technologies could contribute to organize effective and efficient international collaborations to fully utilize this opportunity.

In addition, molecular biological information of subject NHPs in the natural environment (i.e., population genomics, epigenetics, microbiome, nutrition isotope, etc.) can be collected, and correlated with above *in situ* socio-behavioral characteristics of individual animals, to tracking mechanisms of ecological and social variations. Patterns of molecular expression can be measured through the analysis of a variety of discharged bio-samples (feces, saliva, and hair) (Sawaswong et al., 2020), or of exhalation by laser spectroscopy (Selvaraj et al., 2020). Close collaborations with researchers and local personnel (such as staffs of natural parks at NHP habitat) to design the observation system and sample collection, as well as to maintain and operate sensing devices at the filed sites are mandatory to make this infrastructure functional.

Finally, at the physiological and neural levels, a number of novel tools can be used. For example, ultra-thin film bio-sensors attached to the body surface (Nishinaka et al., 2021) and a number of emerging technologies allowing measurements of brain activities through embedded wireless multi-electrode and electrocorticography techniques (Matsuo et al., 2011; Ando et al., 2016) or miniature PET (positron emission tomography) technologies (Schulz et al., 2011) could be utilized. Although these investigations would only offer correlational information and not controlled causal pathways, a number of dynamical networks techniques (Bramson et al., 2019; Aihara et al., 2022) can be applied to detect plausibility of multi-level causal pathways (Stokes and Purdon, 2017).

The hypothesized causal pathways between variables obtained in *Natural Labs* at multiple level of analysis (from behavioral to molecular) can be useful to design controlled experiments in captivity with primates that are hosted under traditional laboratory facilities (Figure 2Ba). In addition, basic elements of these pathways can be investigated under traditional laboratory settings in non-primate animal models, with the goal of inquiring into potential conserved mechanisms present across species (Figure 2Bb,c). In this way, *Natural Labs* (with both *in situ*- and *ex situ*-, to be depicted below) can constitute a bridgehead between classical laboratory and wildlife research approaches (Figure 2B): the multilevel investigations executed on primate groups in natural(istic) settings would provide results and findings to inquire into NHP and non-primate models under controlled conditions in classical captive experiments.

#### *Ex situ* Nature-in-Lab

Above *in situ* Lab-in-Nature is not adapted for neuroscience experimental interventions. A compatible and complementary laboratory setup that simulates (perhaps by being located close to) natural environment and house minimal numbers of NHPs (namely, *ex situ* Nature-in-Lab) could be established to allow longitudinal scientific investigations with minimally invasive neuroscience techniques. In *ex situ* Nature-in-Lab, scientists can study the mechanistic basis of various cognitive functions; perform, along the developmental lifespan of the primate individuals large-scale genetic, epigenetic and metabolite screens; interrogate circuit-level processes of mental functions; and keep tracking of the social behavior of various individuals constituting a primate population group.

In *ex situ* Nature-in-Lab, scientists can collect a large number of heterogeneous data and conduct analysis of multilevel information (from genetic profile to neuronal circuitry, to social behavior), through large-scale investigation of brain, body, cognitive, and behavioral traits. This has the potential to enable systematic characterization of molecular, cellular and circuit-level landscapes of the primate brain and behavior across development and context.

Similar research settings have been utilized in the last decades for testing primate cognitive and behavioral abilities through Computerized Test Systems, which allow animals to live with their social group and to enter some test stations at some time points of the day (Perdue et al., 2018). However, experimental tests in these spaces rarely include neural and molecular investigations; cognitive, neural, and genetic analyses mostly pertain to different laboratories that study biological or imaging samples. In contrast, *Natural Labs* should be intended as spaces where it is possible to train novel, minimally invasive techniques in complex environments, with the aim to collect information that are not possible to obtain in classical laboratory settings, therefore factually being a step forward in the type of information available for research.

Again, like *in situ* Lab-in-Nature, close collaboration with researchers and husbandry personnel is important to promote maximum scientific potential of the *ex situ* Nature-in-Lab. That is, ways to maintain colony in this setup is a part of experimental design and collecting quality scientific data can be completed through daily husbandry activities. Also, collected data could be made accessible real-time on-line, *via* internet, from laboratories located remotely world-wide in which scientists are interested in further analyses of their interest. Hotel space might be associated with *ex situ* Nature-in-Lab for occasional site visits for such remote scientists whenever physical activities are required on site, and perhaps also to make comparative investigations in relation to closely located complementary *in situ* Lab-in-Nature (Tramacere and Iriki, 2021).

*Natural Labs* with novel technologies would become world’s hub of laboratories conducting novel research paradigms. In order for such *Natural Labs* to become a bridgehead between different research approaches, conceptual foundations need to be established to defend following criticisms from mind-sets of current experimental paradigms. Objections could range from lack of experimental sessions, lack of reproducible results under strictly controlled conditions (e.g., fixed head recordings with operantly conditioned tasks in identical experimental environment), or difficulties for applying methods to record neural activity. Importantly however, the present proposal does not aim to completely substitute, but to initially complement existing primate laboratory research. Therefore, neuroscientists can still have the possibilities to conduct primate experimentation in a classical laboratory setting when it is necessary to do so.

On the other hand, classically positioned primatologists may object that is aberrant to violate the life of the animals hosted in semi-natural environments or in the wild. We agree this is a fundamental issue that needs to be addressed through careful discussions. It seems obvious, however, that only a limited number of observations and experiments should be made possible in NHPs living in natural conditions, carefully considering only measures for minimal invasiveness and disturbance, which are very much in accordance with the widely accepted welfare rules in primates.

In the next section, we will delve deeper in the cost-benefit trade-off that this novel strategy of primate experimentation would require, both at the ethic and scientific level.

## Harmonization of cost and benefit trade-offs

### Ethical balance

Natural Laboratory Complex (combinations of *ex situ* and *in situ Natural Labs*) fulfills current ethical standards (CIOMS-ICLAS, 2015) but also provide increased ethical conditions for primates. This is because, on our proposal, researchers of *in situ* Lab-in-Nature would team up with wild-life conservation sectors, and of *ex situ* Nature-in-Lab work together with husbandry team, to ensure obtained data to represent primates’ ecological characteristics, whereby incorporate into extant animal experimentation frameworks (Figure 3) has embedded within it the means to merge formerly segregated wildlife and laboratory research.

From a strictly primate welfare perspective, housing NHPs in natural-like environments, such as *ex situ* Nature-in-Lab, would relief at least some of them from most of the current costs they are paying for neurobiological research, such as being removed and bread distant from their natural and social environment, transported, and subjected to the living conditions of the facilities.

This proposal would be suitable only for the primates subjected to neurobiology studies in the labs, which utilize about the 19% of the total of NHP used in research (Carlsson et al., 2004). However, its potential benefits should not be underestimated. In fact, according to the report of United States Department of Agriculture 2017, a consistent number of primates are held by facilities but not used in any experimental protocols (Grimm, 2020).

This would imply that a minor number of NHPs could be housed in facilities in the future for the scope of behavioral and neurobiological research and produce a situation in which it would be humans who move to study primates, instead to move primates in order to be studied by humans. This is in line with an interpretation of *welfare*, which not only aims to avoid or minimize pain and adverse effects, but also to maximize well-being, through the implementation of environmental enrichment and the promotion of positive elements of comfort and security.

Another benefit of the *Natural Labs* proposal would derive from its possible effects in terms of primate welfare and conservation. According to our proposal, research infrastructures would be established in the wild (*in situ* Lab-in-Nature) and would provide a research basis for neuroscientists and molecular biologists, other than for classical primatologists. Implementing *Natural Labs* would thus have the consequence to increase the amount of field research, and the amount of information related to primate individuals who live in the wild that can be utilized for scopes that could benefit primate welfare in the wild.

Specifically, monitoring the conditions and changes of primates living in the wild may have positive influences on the enterprise associated to wildlife conservation and protection against extinction risks. Because of the documented sudden leaps in aberrant ecosystem behavior, wild populations of NHPs become increasingly susceptible to stochastic genetic, demographic changes, new infectious diseases, and destructive infestations of invasive insects. A detailed understanding of animal biology, ecology, life history, behavior, habitat needs, evolutionary flexibility, and phenotypic plasticity is necessary for promoting their conservation, and preventing extinction dangers (Estrada et al., 2017).

The protection of non-human life and ecological processes necessarily pass through an opportune knowledge of animal and habitat conditions. Therefore, large-scale molecular and behavioral analyses in wildlife through the establishment of research infrastructure can be utilized for tracking extinction dangerous in primate species, their level of distress and other indicators of wellness (Paquet and Darimont, 2010). In addition, by inquiring more in details NHP wildlife behavior, through focus observations or the use of tissue-sample analysis, it is also possible to acquire information that are critical for reproducing naturalistic settings for NHP living in natural parks. Knowledge of the behavior of the animals in the wild it is in fact critical to understand their needs and necessity while they live in semi-natural environments.

Non-human primate living in naturalistic settings may further offer the possibility to investigate a range of naturally occurring dysfunctions in NHPs. In fact, primates show inter-individual variability and differential susceptibility to mental diseases, which can be inquired in order to understand cognitive mechanisms and functions. An example regards the spontaneous development of autistic- or depressive-like phenotype in macaque monkeys (Yoshida et al., 2016). When sample sizes are sufficient, the observation of social behavior in monkeys is likely to be useful in latent variable models that combine indicators of various psychopathologies across multiple levels of analysis (Maestripieri and Lilienfeld, 2016). These cases may constitute precious source of information for biomedical research through medical device testing, drug development and imaging technique refinement.

### Socioeconomic balance

*Natural Labs* might appear costly and hinder continue running on-going experimentations under currently existing academic funding schemes. However, introduction of latest AI, IT, and VR technologies (see section “Natural laboratory complex”) offer *Natural Labs* an opportunity for collaborations with various research sectors, including non-academic sectors, such as various industries and regional economy, to attain autonomous financial foundations for its shared and mutual benefits still yet securing academic independence of research communities.

Such models could include “Real-time 3D Digital Zoo” in entertainment businesses, or the one by governmental supports allowing visitors to the educational programs to be settled in the *Natural Labs* within a traditional zoo/safari perspective (see also Box 3 for potential schemes of cross-sector collaborations). Hence, these self-supported *Natural Labs* would enable researchers in countries far from NHP natural habitat, with minimal expenses for participation, to access data remotely at home laboratories, and hotel space at *Natural Labs* offers conductive possibilities for studying various natural behaviors of NHPs on site.

**BOX 3 Development of sustainable business–academia–government collaborations.**

Biodiversity education for the *Natural Lab’s* local site population is paramount to preserve the natural equilibrium of native ecosystems with tourism providing cash input. Online interactive datasets could be created to construct a “Real-time 3D Digital Zoo” as an *ex situ* global research center located adjacent to the *Natural Labs*. Technological development for this research will contribute to environmental protection with future profitability. Data comprising “Real-time 3D Digital Cloud Zoo” will be shared between the scientific community and various business models.

A novel neurobiological principle established through the *Natural Labs* characterizes human gene-culture co-evolution through environmental interactions. Novel non-invasive technologies (perhaps AI and VR-based) developed through these studies will fulfill the requirements of primate research ethics and wildlife conservation. This will also emphasize how humanity can evolve as a part of holistic ecosystems and allow us to envision how our biodiversity should be developed in the future within this ever-changing world.

This research setup can immediately detect “skeleton” behaviors of monkeys for motion analyses, together with rough geometry of the landscape for interactions with the environment at each *Natural Labs* site. While this data will comprise core information to establish the “Digital Cloud Zoo,” it would be ideal if supports by businesses and industries to create further sophisticated appearances of the animals and landscape suitable for exhibition to the public. Also, initial setup is limited to only a small part of the nature for research, and thereafter industry and government-supported collection of data covering larger areas of the local natural habitat will promote more systematic and wider range of knowledge eventually contribute to preservation of the species across the habitat.

Extension of these scheme desires to create mechanisms that reveal basic data and share findings with the worldwide research community (this is of particular importance to establish novel global ethical standards for primate research that contribute to future studies for human mental welfare). Procedures enabling this research-oriented “Digital Cloud Zoo” will share common IT and cloud technologies for exhibition at museums run by local governments, which can further promote this research through entertainment and businesses that can reach global audiences.

Thus, it would be ideal to institute mechanisms that are equally beneficial for research, government, and industry/business partners. This could be in turn contribute to scheme governmental policies to plan a new strategy that will include ecotourism and employment of local people to serve as a natural resource for the local countries.

Another benefit of this approach is related to those countries endowed with natural habitats for NHP, such as Japan, India, or Southeast Asian countries, which suffer from increasing conflict between humans and feral monkeys over the last several decades (Dittus, 2012). NHPs become pests because they pilfer food and water near human habitation. Artificial feeding leads to changes in monkey behavior, resulting in overpopulation of aggressive monkeys. Exterminating a large number of these monkeys is unethical according to religious beliefs, and also according to the welfare standards developed in several countries.

Further, castration and trans-location practices are overly expensive and laborious because they require specialized personnel and long-lasting procedures. In Japan, for example, several monkey parks are spread throughout the territory and are open to visitors. In Singapore, although not specifically for primates, there are many Safaris where animals live in wild-like environment and are managed by caretakers under international standards of animal welfare. Revenues to *Natural Labs* from above business models could create similar mechanisms for social visiting and education benefits, and these animals can constitute a further source for studying inter-individual variability at the behavioral level and analyzing various biological samples.

### Legal balance

A novel factor of the present proposal of *Natural Labs* which requires additional consideration is the management of the data acquired here are shared globally across multiple parties under having potential conflict of interest. This is an essential requisite due to its geographically biased natural distribution of NHPs by ecological limitation, which is complemented by web-based international data sharing among various sectors. While such situation secures transparencies to fulfill ethical and scientific standards of subjected experimentations, at the same time risks protection of intellectual properties, originality and privacy of ideas that might belong to parties (individuals, groups, institutions, nations, etc.) who participate in the project.

Rules to legally harmonize conflicting interests among such parties yet need to be established [Iriki quoted by Marx (2016)]. Such harmonization should include legal measures of, (a) how to ensure priority of shared data among international academic members across borders of national sovereignty and security, (b) how to protect intellectual properties, if any emerged, upon data sharing among industry, academia, and other relevant sectors, (c) how to manage, or restrict, balances between scientific accuracy/significance and public stakeholders’ interests/perception especially when related with socioeconomical aspects or public policies. Considering novel aspects of presently proposed *Natural Labs* paradigms, we just exemplify these, among many other potential issues, for future considerations to be incorporated into international public policy frameworks, in addition to digital technologies for managing general security of data transferred *via* internet environments.

## Conclusion

We have proposed novel strategies for primate experimentation with potential ethical, scientific, and economic benefits. Specifically, we have proposed the establishment of *Natural Labs*, as combination of indoor-outdoor structures where to conduct cognitive neuroscience investigation in naturalistic environments with various species of monkeys.

*Natural Labs* are thought to studying various aspects of mind, body, and behavior in the primates, where the goal is generalization of this knowledge to understand the mechanistic bases of human mental functions and vulnerability to disorders. Research in primate *Natural Labs* may be beneficial for (1) enhancing scientific validity, through the study of the embodied and socially situated aspects of mental functions in primates, in longitudinal fashion and ecological settings, (2) providing naturalistic settings and wild-like environment for primates, thus increasing primate welfare, (3) reducing the monkey-human conflict in areas where monkeys are becoming pest and allowing data collection that can be instrumental to primate conservation.

This proposed novel strategy of primate experimentation is of particular importance for understanding mechanisms of human mind, in its functional and dysfunctional manifestation, because human beings are tropical primates and have expanded their habitat by explosive mental development (without physical changes) in the last tens of thousands of years (Iriki et al., 2021).

Although the realization of *Natural Labs* is in accordance with current ethical framework, it subtly incentivizes an enrichment of the current understanding of animal ethics. *Natural Labs* can enrich primate welfare with a *novel perspective*: primates have the potential for living and/or interacting collaboratively with humans and exchange needs and requirements with them. This species differences affect the relationship we can establish with and the value we assign to them: beyond the protection of primates according to their capacity to experience pain, the approach of *Natural Labs* would actively increase primate welfare.

Realizing *Natural Labs* for primate research and implement correlated investigative practices could require the establishment of updated ethical guidelines, which can regulate from the one hand, the interactions between human and NHPs, and from the other hand, rules and regulations between various research centers, laboratories and researchers. Although the aim of this manuscript is not to propose these guidelines but to generate discussion, we hope that this perspective can constitute a fruitful addition for achieving further progress in primate experimentation and welfare.

## Author contributions

AI and AT contributed to conception and design of the study. Both authors contributed to manuscript revision, read, and approved the submitted version.
